# Neurofibromatosis type 2 tumor suppressor protein is expressed in oligodendrocytes and regulates cell proliferation and process formation

**DOI:** 10.1371/journal.pone.0196726

**Published:** 2018-05-01

**Authors:** Andrea Toledo, Elena Grieger, Khalad Karram, Helen Morrison, Stephan L. Baader

**Affiliations:** 1 Institute of Anatomy, Anatomy and Cell Biology, Bonn, Germany; 2 Laboratorio de Cultivo de Tejidos, Sección Biología Celular, Facultad de Ciencias, UdelaR, Montevideo, Uruguay; 3 Institute for Molecular Medicine, Johannes Gutenberg University Mainz, Mainz, Germany; 4 Leibniz Institute for Age Research, Fritz Lipmann Institute, Jena, Germany; Instituto Cajal-CSIC, SPAIN

## Abstract

The neurofibromatosis type 2 (NF2) tumor suppressor protein Merlin functions as a negative regulator of cell growth and actin dynamics in different cell types amongst which Schwann cells have been extensively studied. In contrast, the presence and the role of Merlin in oligodendrocytes, the myelin forming cells within the CNS, have not been elucidated. In this work, we demonstrate that Merlin immunoreactivity was broadly distributed in the white matter throughout the central nervous system. Following Merlin expression during development in the cerebellum, Merlin could be detected in the cerebellar white matter tract at early postnatal stages as shown by its co-localization with Olig2-positive cells as well as in adult brain sections where it was aligned with myelin basic protein containing fibers. This suggests that Merlin is expressed in immature and mature oligodendrocytes. Expression levels of Merlin were low in oligodendrocytes as compared to astrocytes and neurons throughout development. Expression of Merlin in oligodendroglia was further supported by its identification in either immortalized cell lines of oligodendroglial origin or in primary oligodendrocyte cultures. In these cultures, the two main splice variants of *Nf2* could be detected. Merlin was localized in clusters within the nuclei and in the cytoplasm. Overexpressing Merlin in oligodendrocyte cell lines strengthened reduced impedance in XCELLigence measurements and Ki67 stainings in cultures over time. In addition, the initiation and elongation of cellular projections were reduced by Merlin overexpression. Consistently, cell migration was retarded in scratch assays done on *Nf2*-transfected oligodendrocyte cell lines. These data suggest that Merlin actively modulates process outgrowth and migration in oligodendrocytes.

## Introduction

The neurofibromatosis type 2 (NF2) protein Merlin is a tumor suppressor protein known to be expressed in supportive tissue of the peripheral nervous system and in a variety of other organs of the body [1]. More recently, it was shown that Merlin is also expressed in the central nervous system, in particular in neurons, neural precursor cells and astrocytes [2–6]. Inactivation of the *Nf2* gene in the germ line leads to the development of benign schwannomas, meningiomas and gliomas, tumors that endanger the patient by compressing important structures of the nervous system [7–10]. Merlin is also inactivated in sporadic tumors outside the nervous system, such as mesotheliomas, thyroid and skin cancer [11].

Merlin is a member of the ERM (ezrin, radixin, moesin) family, known to interact with the actin cytoskeleton [12]. As with other members of the ERM family, Merlin is concentrated in the cytoplasm and nucleus where actin filaments dynamically rearrange to form lamellipodia, filopodia, microspikes or the cleavage furrow [8]. By supporting these functions Merlin serves as a link between the plasma membrane and the actin cytoskeleton through regulating Rac-PAK, Ras-ERK, Raf-MEK-ERK, PI3-Akt, or FAK-Src pathways, thus impacting on membrane trafficking and cell signaling [13–20]. All these signaling components are active in the central nervous system arguing for a potential role of Merlin in regulating cell proliferation, cell adhesion, process formation, and/or cell migration.

The *Nf2* gene is organized in 17 exons that code for two main isoforms distinguishable by the C-terminal domain. Merlin isoform 1 is coded by exons 1 to 15 and 17 and has 595 amino acids; isoform 2 has 590 amino acid residues and results from the introduction of a stop codon in the spliced exon 16 [8]. Thus far, 10 isoforms with distinct spatial and temporal expression patterns have been described [21–23]; however, their function remains unclear.

Merlin was shown to be clearly expressed in the peripheral nervous system and in neurons and astrocytes of the central nervous system [1,9,24]. Immunohistochemical studies have shown that Merlin is widely expressed in coarse cytoplasmic granules in both glia and neurons in the central nervous system [25]. Astrocytes and neurons react to changes in Merlin expression levels by altering cell morphology [3,26,27]. However, evidence of its presence in oligodendrocytes is much more limited and confined to only a few studies. Initial *in situ* hybridization studies could not detect *Nf2* mRNA in the white matter [28]. In contrary, immunohistochemistry revealed small clusters of NF2-positive granules around oligodendroglial nuclei [7]. In addition, transcriptome analysis revealed significant expression of NF2 in purified oligodendroglial cells [29]. No detailed analysis has been performed to date, possibly due to the fact that mutations in the *Nf2* gene have thus far been related to the development of schwannomas, meningiomas and gliomas—but have not been described in patients harboring oligodendrogliomas [2,30,31].

In an effort to enhance our understanding of the role of Merlin in oligodendroglial cells, we studied its presence in developing and mature oligodendrocytes in brain tissue. We also investigated its presence in mouse oligodendrocytes and in different oligodendrocyte cell lines. By means of stable Merlin overexpression in oligodendrocyte cell lines, we also evidenced the tumor suppressor effect of Merlin and its ability to regulate proliferation and process formation/migration.

## Materials and methods

### Animals

All animals used in this work were housed under constant temperature and humidity conditions on a 12 h light/dark cycle, with access to food and water *ad libitum*. Animals older than six weeks were classified as adults. NG2-EYFP mice, which carry a YFP fluorescent protein gene instead of a NG2 gene, are healthy and fertile and do not show any obvious phenotype [32]. They were kindly provided by Dr. J. Trotter (Institute of Molecular Biology, Mainz, Germany). Mice for cell cultures were obtained from breedings of C57Bl6/J mice (Janvier Labs, France). All mice were handled in strict adherence to local governmental and institutional animal care regulations and were approved by the Institutional Animal Care and Use Committee (Landesamt für Natur, Umwelt und Verbraucherschutz Nordrhein-Westfalen) under the permission number Az 84–02.05.20.13.121. For mouse anesthesia, a mixture of Ketamin/Rompun was intraperitoneally injected, and mice were sacrificed by cervical dislocation after anesthesia.

### Immunohistochemistry

To assess Merlin localization on nervous tissue, adult and nine day old mice (P9 mice) were transcardially perfused using Ringer´s solution, followed by 4% paraformaldehyde in phosphate buffered saline (PBS). Brains were dissected and postfixed for 6 hours. Sagittal sections were performed using a vibratome (VT1000 S; Leica, Wetzlar, Germany). Sections were heated in 10 mM sodium-citrate buffer pH 9, at 80°C for 30 minutes, for antigen retrieval. After cooling, sections were incubated in 2% nonfat dry milk for 30 minutes and then in 0.5% Triton X-100 for 20 minutes. Blocking of unspecific sites was done by incubation in 0.2% gelatin, 2% goat serum in PBS, at room temperature for 2 hours. Primary antibodies were incubated at 4°C overnight. The following antibodies were used: anti-CalbindinD-28K (rabbit polyclonal, 1:2000 Swant CB-38a, Marly, Switzerland and mouse monoclonal 1:1000 Sigma-Aldrich (clone CB-955), Munich, Germany), anti-Merlin (rabbit polyclonal antibody, A-19 (sc-331) and C-18 (sc-332), 1:50; Santa Cruz, Heidelberg, Germany), anti-Olig2 (mouse monoclonal antibody, 1:500; Millipore (AB9610), Darmstadt, Germany), anti-MBP (mouse monoclonal antibody, 1:500; Chemikon (MAB384), Schwalbach, Germany). After three PBS washes, sections were incubated in the secondary antibody solution (Alexa Fluor 488 and 546 conjugated goat anti-mouse and anti-rabbit antibodies A-11001, A-11008, A-10036, A-10040, 1:500, Invitrogen, Darmstadt, Germany) at room temperature for 2 hours. After PBS washing, sections were embedded in Mowiol.

### Cell culture

OLN93 mouse oligodendrocyte cell line was kindly provided by Dr. C. Richter-Landsberg (Oldenburg University, Germany). This permanent cell line is derived from spontaneous transformation of rat glial cultures and represents typical oligodendrocyte protein expression such as myelin basic protein, myelin associated protein [33] and Olig-2 (as shown here). SC4-immortalized *Nf2*^-/-^ primary Schwann cells were derived from Nf2^-/-^ deficient mice [19,34]. They thus contain a *Nf2* gene missing exon 2 and 3 which results in an unstable and, if at all, truncated and non-functional protein version [35,19]. The RT4-D6PT2 schwannoma cell line was obtained from the European Collection of Animal Cell Cultures (Salisbury, United Kingdom). This cell line was originally derived from a N-ethyl-N-nitrosourea (ENU) induced rat peripheral neurotumor and was used as a model cell line for Schwannoma [36,37]. The TC620 human oligodendroglioma cell line was a gift from Dr. A. Glassmann (Life Science Incubators, EPN-Technology, Germany). TC620 cells were cultured from human oligodendroglioma tissue and show oligodendroglial like ganglioside expression levels and pattern [38,39]. All cell lines were maintained in DMEM medium supplemented with 10% heat inactivated FCS, 100 U/ml penicillin and 100 μg/ml streptomycin. The extraction of primary oligodendrocytes (CG4) was performed according to the protocol of Bottenstein and Sato, 1979, modified by Louis et al., 1992 [40,41]. Briefly, cells were harvested by dissecting 4–6 forebrains from newborn heterozygous NG2-EYFP mice [32]. Animals were decapitated, the hemispheres dissected and dissociated in 0.25% trypsin-EDTA. The cell suspension was plated on poly-L-lysine (PLL) coated dishes, using standard DMEM medium supplemented with 10% fetal calf serum. After one week of incubation, the cultured primary oligodendrocytes were rinsed off from the underlying astrocyte layer and plated on fibronectin-PLL-coated dishes filled with N1-medium containing glutamate (2 mM), transferrin (50 μg/ml), insulin (5 μg/ml), putrescine (100 μM), progesterone (20 nM), selenium (30 nM), biotin (10 ng/ml), Pen/Strep (100x) dissolved in DMEM and supplemented with a conditioned supernatant of B104 neuroblastoma cells. After a further 5 days of incubation, cultured primary oligodendrocytes were fixed or harvested for Western blot. All reagents were obtained from Life Technologies (Darmstadt, Germany) or Sigma-Aldrich (Munich, Germany).

### Immunocytochemistry

For immunocytochemistry, cells were fixed in 4% paraformaldehyde in PBS for 10 minutes at 37°C. Plates used for Ki67 stainings were then incubated in 10 mM Na-citrate (pH 9.5) at 80°C for antigen retrieval. All cultures were then processed the same way: they were permeabilized with 0.1% Triton X-100 for 3 minutes, non-specific antibody binding sites were blocked using a mixture of 1% bovine serum albumin and 10% goat serum for two hours, incubations with the primary antibodies were done in blocking solution at 4°C, and carried out overnight. The following primary antibodies were used: anti-Merlin (clone A-19 and C-18, 1:50, Santa Cruz Biotechnology, Heidelberg, Germany; or HPA003097, 1:100, Atlas Antibodies, Bromma, Sweden), anti-Olig2 (1:500; Millipore, Darmstadt, Germany), anti-GFP (A11122, 1:500, Thermo Fisher, Schwerte, Germany), anti Ki67 (15580, 1:500, Abcam, Cambridge, UK), and anti phospho-Histone H3 (pH3-Ser10, 06–570, 1:1000, Millipore, Darmstadt, Germany). After extensive rinsing in PBS, cells were incubated with goat anti mouse or anti rabbit secondary antibodies linked to Alexa Fluor-488 and 546 (1:500; Invitrogen, Darmstadt, Germany) for 1 hour. Afterwards, cells were washed in PBS and counterstained with DRAQ 5 (1:1000 in PBS, Biostatus, UK), or phalloidin conjugated to Alexa 546 (1:100 in PBS, Invitrogen, Darmstadt, Germany) and Hoechst 34580 (1μg/ml in PBS, Invitrogen) for 20 min. Slides were mounted in Mowiol.

### Quantification of proliferating cells

Proliferation of oligodendroglial cells was analyzed in 24 well plates in which cells were seeded at a concentration of 2x10^4^ cells per well when cultured for up to 48 hours, and at a density of 1x10^4^ cells per well when incubated for more than 48 hours. After seeding, cells were grown for 24, 48, 72, and 96 hours, fixed, stained for Ki67 or pH3 and counterstained with Hoechst 34580. Images were taken with a Leica DMIRE2 inverted microscope equipped with a DFC350 camera using defined imaging parameters for each fluorophore. In order to avoid bias by the experimentators, six images were taken along the diameter of each well only selecting for regions which showed comparable cell densities (subconfluent at the beginning of culturing and confluent after 96 hours in culture). Then background was subtracted in ImageJ using a rolling ball radius of 50 pixels. Frequencies of pixels with intensities larger than 20 (out of 255) were recorded for all images and summed up to get numbers of pixels positive for Ki67 or Hoechst, respectively. Ki67 positive pixels were then related to Hoechst positive pixels and thus serves as a measure of Ki67 expressing cells. Each experiment was done in triplicate. We thus obtained a total of 12 images per time point and cell line. In order to demonstrate a time dependent effect of Merlin overexpression on cell proliferation, we combined data obtained on days two to four and compared them to data obtained at day 1. Equal variances of ratios were demonstrated by Levene’s test. Significance was tested by one-way ANOVA (for day 1) and two-way ANOVA for days 2–4 (ns not significant, *p < 0.05, ***p < 0.001). Data analysis was done using R [42].

### Western-blot

Subconfluent cell line cultures were trypsinized, washed in PBS and lysed in RIPA (Radio-Immunoprecipitation Assay) buffer supplemented with protease inhibitors (Pefabloc, Sigma-Aldrich, Munich, Germany). Protein concentrations were determined with BCA^TM^ kit (Thermo Scientific, Darmstadt, Germany). Samples were loaded and separated by SDS-PAGE in a 10% polyacrylamide gel, before being transferred to a polyvinylidene fluoride membrane (Millipore, Darmstadt, Germany) with a semidry transfer system (Nova Blot, GE Healthcare, Munich, Germany). Unspecific binding sites on the membrane were blocked with 5% milk resuspended in 0.1% Tween-20 in PBS for two hours. Primary antibodies were diluted in blocking solution and incubated overnight at 4°C (anti-NF2 clone C-18, 1:500, Santa Cruz, Heidelberg, Germany; anti-phospho S518-Merlin 1:1000, and anti-cyclophilin B 1:4000, Abcam, Cambridge, UK). After three 10 minutes’ washes in 0.1% Tween-20 in PBS, membranes were incubated with the secondary antibody diluted in blocking solution for two hours at room temperature (goat anti-mouse or rabbit HRP, 1:8000, Dako, Hamburg, Germany). Immunoreactive bands were visualized using SuperSignal® West Pico chemoluminescent Substrate (Thermo Scientific, Darmstadt, Germany). Each experiment was repeated at least three times. Optical density measurements of whole bands were performed using the Image J (NIH) Gel analysis plugin. Results were normalized using cyclophilin B. Results are shown as mean ± standard error of the mean (SEM). Statistical analysis was completed using Student t-test, *p < 0.1, **p < 0.05, ***p < 0.001.

### Reverse transcription PCR analysis

Total RNA was isolated from adult mouse brains and from cell lines by homogenization in Trizol (Life Technologies, Darmstadt, Germany). cDNA was synthesized from 1–2 μg of RNA by reverse transcription using Superscript III polymerase (Life Technologies, Darmstadt, Germany), in accordance with the suppliers’ instructions. Merlin isoforms were amplified using the primers listed in Table 1.

**Table 1:** Primer sequences.

Species	Amplicon	Primer sequence (5´-3´)	Amplicon length (bp)
**Mouse**	Exon 1–6	AAGCAGCCCAAGACATTCAC	
**Mouse**	Exon 1–6	CGCATACCAAGCCGTAATTC	530
**Mouse**	Exon 4–13	CGTCCGTGCTCTTGGCGTCAT	
**Mouse**	Exon 4–13	CGTCCGTGCTCTTGGCGTCAT	1030
**Mouse**	Exon 12–17	AGGTGCTGGAGGCTGAAGTG	
**Mouse**	Exon 12–17	TCAGGCCCATCCTGTTGGTG	566
**Rat**	Exon 1–6	AAGCAGCCCAAGACATTCAC	
**Rat**	Exon 1–6	TCTCCAGGTCCTGAGCTATC	587
**Rat**	Exon 4–13	GCACAAGCGAGGATTTTTAG	
**Rat**	Exon 4–13	GGTGGGCTTGGTGGCGATTTC	1030
**Rat**	Exon 12–17	CTGAAGTGCTGGCATTGAAG	
**Rat**	Exon 12–17	AAGCCAGCCCTCTACTGATG	628
**Human**	Exon 1–6	GGGCTAAAGGGCTCAGAGTG	
**Human**	Exon 1–6	TCG GTGCTCTGCGTACCAAG	587
**Human**	Exon 4–13	TGGCTTCTTACGCCGTCCAG	
**Human**	Exon 4–13	TGGGCTTGGTGGCAATCTCC	1030
**Human**	Exon 12–17	AGCAGGAAATGCAGCGCATC	
**Human**	Exon 12–17	AGAAGGCCACTCGGGACTTG	628

Primer sequences are given in pairs in 5’ to 3’ direction for each amplicon in each species. The calculated amplicon length is given in base pairs.

### Cell transfection

For stable transfection of Merlin in cell lines, the plasmid pcNf2hflag containing a CMV promoter and a neomycin resistance cassette was used [19]. An empty vector pcDNA3.1 was used as a control. Cell lines were transfected using Lipofectamine LTX (Invitrogen, Darmstadt, Germany), again as per manufacturer´s instructions. For preparation of stable cell lines the neomycin resistant gene of the vectors was used, and cells were selected with Geneticin (G418, 600–1000 μg/ml, Life Technologies, Darmstadt, Germany).

### Measurement of cell impedance by XCELLigence

The microelectronic biosensor *XCELLigence* Real Time Cell Analysis System (RTCA, Roche Diagnostics GmbH, Mannheim, Germany) was used to measure the resistance introduced to an electric field by growing cells over time. This is performed by microelectrodes attached to the bottom of the 96 well plates which measure impedance as a dimension-less cell index through the plate, which is proportional to the number and pattern of attached cells. First, the background impedance was measured according to manufacturer´s instructions with 100μl of standard medium. To determine the optimal seeding density of cells on the RTCA system, different cell numbers (5 x 10^3^, 1 x 10^4^, 2 x 10^4^, and 4 x 10^4^) were plated with 100μl of culture medium. Impedance was measured every 5 minutes during the first 5 hours (adhesion phase) and every 15 minutes for 100 hours (proliferation phase). The impedance was expressed as an instrument intrinsic parameter—the cell Index (CI). Based on the slope of the CI curves obtained and the time when impedance reached maximum values, we have selected 4 x 10^4^ *control-*transfected and *Nf2-*transfected cells for further experiments. Having measured the impedance curves of these cell lines, we calculated the slope of the curves starting from the minimum CI value after an initial cell adhesion phase, to the maximum of the CI curve. The slopes obtained from growth curves of *control*- and *Nf2-*transfected cells were compared and significance was evaluated using the non-parametric Wilcoxon Rank test. Experiments were conducted in duplicates or triplicates and repeated at least three times.

### Measurement of process outgrowth

Numbers of processes were counted per cell. Only isolated cells and aggregates with less than four cells were evaluated. Measurements were performed on epifluorescent images using Image J (NIH). Process length was evaluated independent of the aggregate size, starting from the cell surface to the tip of a process. Data are presented as mean ± SEM and analyzed by Kolmogorov-Smirnov test. Significance was set at p < 0.05. P values: * < 0.05, ** < 0.01, *** < 0.001.

### Scratch assay

Cells of various cell lines were plated on poly-L-lysine coated plates at high density, enabling the attainment of confluence overnight. Cell-free regions were generated after cell confluence using a sterile pipette tip. Dying or dead cells were removed by changing culture media. Refilling of the scratched area was followed by time lapse imaging taking images every 60 min during the first 10 hours, then every 30 min during the next ten hours (this applies for OLN93 and TC620 cells) and again every 60 min during hours 20 to 30. Since RT4 cells migrated much faster, images were taken every 10 minutes during the 10 to 20 hour phase. For refilling of scratches the cell-free area was measured at six different spots within a 3 cm well plate every 4 hours up to 24 hours using the ImageJ MRI Wound healing tool. Experiments were repeated four times. Results are shown as mean ± SEM. Statistical analysis was achieved using Two-Way ANOVA and Bonferroni post-hoc-test, *p < 0.1, **p < 0.05, ***p < 0.001. To compare mitotic activity during the initial time frame, cells were fixed after 16 hours of the scratch assay and stained for phospho-Histone H3. The staining procedure was done as described above. As a measure of proliferation, phospho-H3 positive cells were related to the total cell number in an area covering 400 μm next to the edge of the scratch to get the mitotic index. In addition, we used time lapse imaging to track single cells. In each view of the time lapse images, we selected up to ten cells and tracked them from the beginning up to the end of the run. Only those cells were chosen which were not encased by other cells during the first 10 hours, and which ran for at least 25 hours. Dividing cells were included by following the path of only one of the daughter cells. Total lengths of paths were compared by Wilcoxon’s rank test.

### Microscopy, image acquisition and statistical analysis

Confocal images of brain sections were acquired with a Leica TCS SP2 laser scanning microscope using 40x 1,25NA and 63x 1,4NA lenses. Epifluorescent and time lapse images were obtained in a Zeiss Axioskop 2 and Leica DMIRE2, equipped with a DFC350Fx camera. Adobe Photoshop (CS and Elements 15, Adobe Systems) and ImageJ (Fiji 1.51, NIH) were used for digital processing of images (linear adjustments of brightness and contrast). Statistical analysis was performed with R [42].

## Results

### Merlin expression in the central nervous system

**Fig 1:**
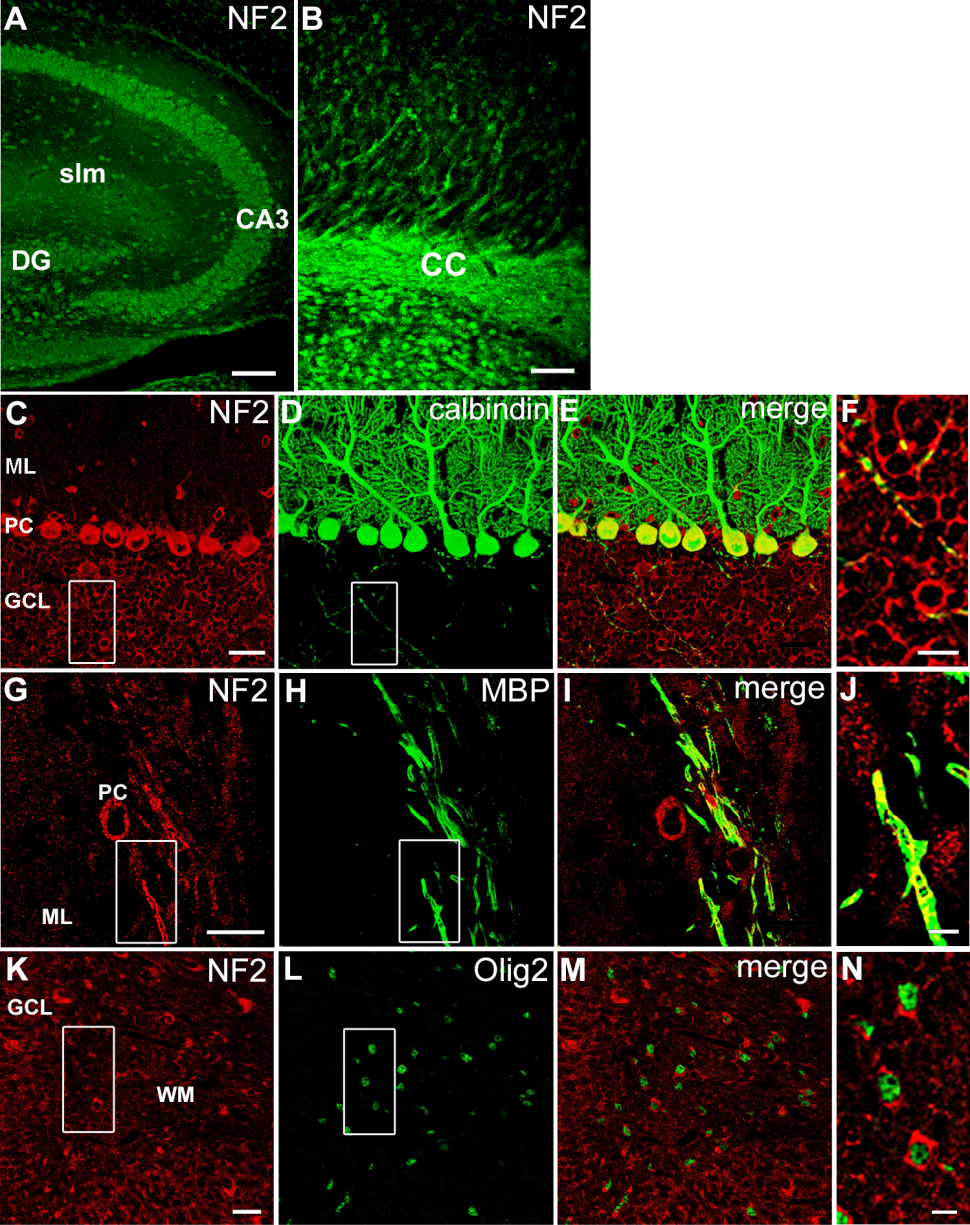
Expression of Merlin in oligodendrocytes of the forebrain and cerebellum. Merlin is present in neuron enriched areas of the hippocampus (dentate gyrus DG and corpus ammonis CA3), but also in the stratum lacunosum moleculare (slm) of the hippocampus (A) and the corpus callosum (cc) of the cortex cerebri (B) of adult mice. Merlin is localized in close approximation to Calbindin (C-F) and MBP (G-J); high magnifications in F, J demonstrate the presence of NF2 in neurons and oligodendroglia. NF2 staining is also present in a subset of Olig2-positive cells of cerebella of nine day-old mice (K-N). Abbreviations: DG: dentate gyrus, cc: corpus callosum, GCL: granule cell layer, ML: molecular layer, PC: Purkinje cells, slm: stratum lacunosum moleculare, WM: white matter. Scale bars: (A) 1000 μm, (B) 25 μm, (C-E, G-I, K-M) 25 μm, (F, N) 10 μm, (J) 5 μm.

In order to analyze the presence of Merlin in oligodendrocytes, we performed immunohistochemical stainings of forebrain and cerebellar sections obtained from adult mice. This staining technique allowed us to colocalize NF2 stainings with known marker proteins for different cell types within the cerebellum avoiding potential misinterpretation of results obtained by promoter driven reporter gene expression in transgenic mice [43–45]. Using NF2-specific antibodies, we found intense immunostaining throughout gray and white matter regions of the brain. To ensure the specificity of the staining, we used an established Merlin-specific antiserum which demonstrated expression of Merlin in different neuron-enriched regions of the brain, such as the hippocampus (Fig 1A) and the cerebellar cortex (Fig 1C and 1G) [3,46]. No such staining could be observed when the primary antibody was omitted (not shown). We previously demonstrated that this staining could be blocked by an antibody specific blocking peptide, and we could also show an increase in the specific signal when overexpressing *Nf2* [3]. White matter staining was particularly evident in the corpus callosum (Fig 1B) and in the arbor vitae of the cerebellum [3]. In order to allocate the Merlin immunosignal to either neuronal or glia processes, we double-stained cerebellar sections of nine day old pups (P9 mice) and adult mice for Merlin and either Olig2 or myelin basic protein (MBP) (Fig 1G–1N). Both proteins are marker proteins of the oligodendroglial lineage. As previously shown, expression was high in Purkinje cells which are specifically labeled by Calbindin antibodies, granule neurons (representing most of the cells within the GCL layer) and the neuropil of all layers (Fig 1C). We also found an evident signal for Merlin allocated around Olig2-positive cell nuclei in the cerebellar white matter of P9 mice (Fig 1K–1N), and we detected NF2 co-localized with MBP-positive fibers in the inner granule cell layer of the cerebellum of adult mice (Fig 1G–1J). This suggests that Merlin is expressed in immature as well as in mature oligodendrocytes. Although clearly discernible, the expression level seemed to be lower in oligodendrocytes as compared to that in neurons.

### Merlin expression in oligodendroglial cells in culture

The *in vivo* findings prompted us to verify Merlin expression in oligodendrocyte primary cell cultures and in cell lines derived from immortalized oligodendrocytes. OLN93 cells are spontaneously transformed cells of primary cultures derived from rat brains [47,33], Oli-neu cells have been generated by transformation of mouse oligodendrocyte precursor cells with the oncogene *t-neu* [48]. Primary oligodendrocyte cultures contain dissociated cells enriched for oligodendrocytes derived from mouse brain [41]. TC620 cells are derived from human oligodendroglioma [38]. The RT4-D6P2Tcell line was established by subcloning of ENU-mutated rat schwannoma cell lines [36]. SC4 Schwann cells are derived from Merlin deficient transgenic mice harboring a Neu oncogene mutation [19]. RT4 and SC4 Schwann cells are already known to express Merlin at moderate levels [19] and were used here as control cells.

**Fig 2:**
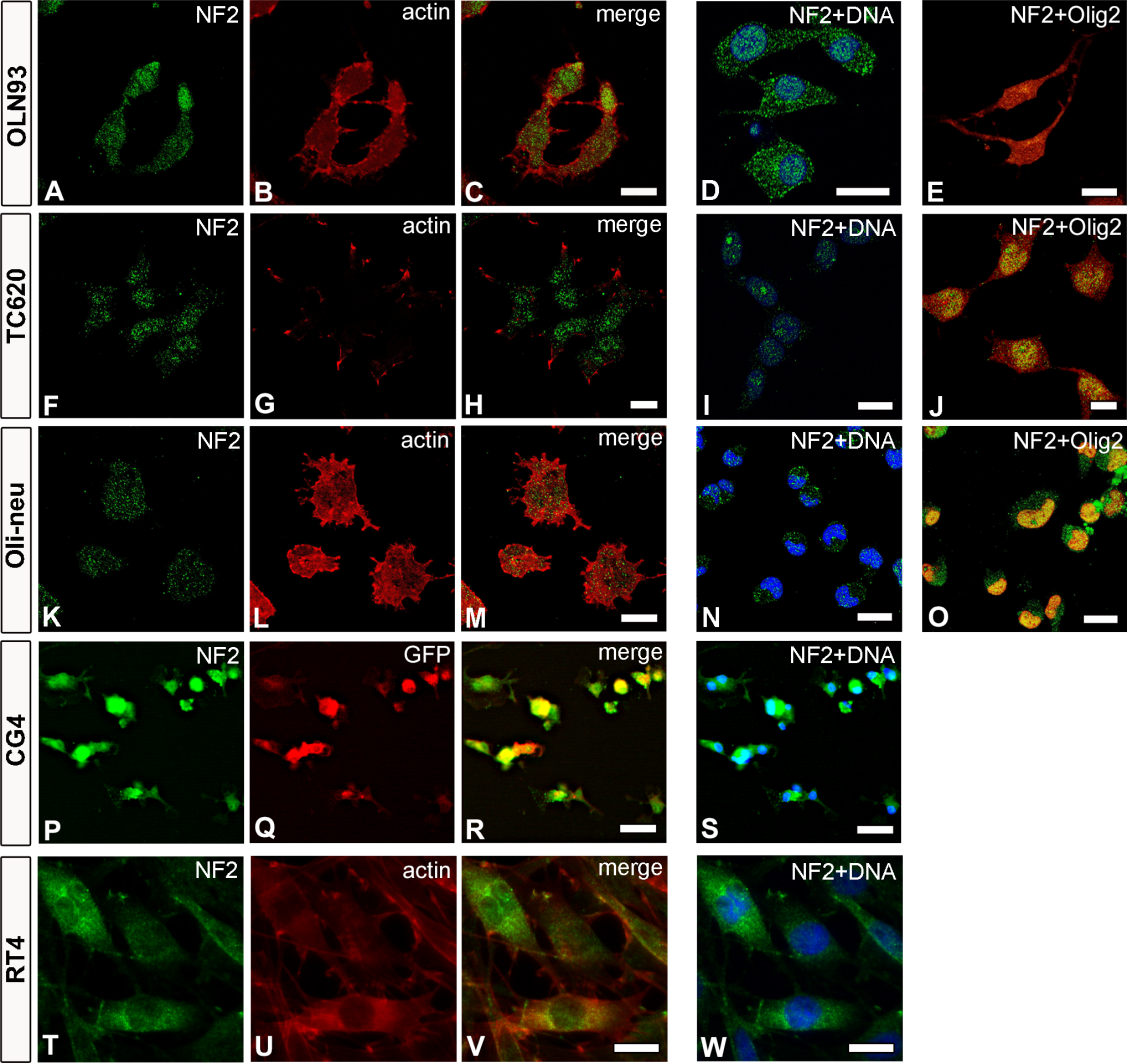
Expression and localization of Merlin in oligodendrocytes. Merlin (NF2) expression is demonstrated by immunostaining and visualized in green color in all cultured cells. Images in A-O represent confocal images of a single plane (using a 63x objective and 1 Airy Unit pinhole size), while oligodendrocyte primary cultures and RT4 cells were taken as epifluorescent images (P-W). Cells were counterstained with phalloidin (B, G, L, U), GFP to show NG2-positive cells (Q), DRAQ5 (D, I, N), Hoechst 34580 (S, W) and Olig2 (E, J, O). Scale bars: 20μm.

Merlin was detected in all cells analyzed by immunofluorescence. In OLN93 cells, Merlin mainly localized in the cytoplasm, and some signal at variable intensity was evident in the nuclei as shown by Hoechst counterstaining (Fig 2A–2D). Thus, Merlin co-localized with Olig2, a marker of oligodendroglial lineage, which is located both in the cytoplasm and in the nuclei of OLN93 cells [49] (Fig 2E). In addition, our results show that almost all of the OLN93 cells showed Olig2 expression, although to a variable degree. In the human oligodendroglioma cell line TC620, Merlin was mainly concentrated in nuclei (Hoechst-positive) and only sparsely distributed in a granular pattern within the cytoplasm (Fig 2F–2J). A comparable localization was found in mouse Oli-neu cells showing Merlin associated with particles located within nuclei (Hoechst staining) and cytoplasm (Fig 2K–2O). Finally, we also detected Merlin in cultured primary oligodendrocytes (cultured primary oligodendrocytes) prepared from knock-in NG2-EYFP heterozygous mice (32). Merlin was highly concentrated in nuclei (DRAQ5-positive) of immature oligodendrocytes, which can be labeled for NG2-EYFP mice using GFP antibodies (Fig 2P–2R). It was also present in the cytoplasm (Fig 2S). In addition, we identified GFP-negative, but Merlin-positive cells which most probably represent astrocytes frequently present in primary cultures [50]. Again, a granular pattern is apparent and, given the epifluorescent imaging, to a comparable extent as shown for cell lines. As described before, RT4 Schwannoma cells contained Merlin in nuclei and in the perinuclear regions of the cytoplasm. Merlin was also present within cell extensions and, it was found in a granular pattern in both cell compartments (Fig 2T–2W).

**Fig 3:**
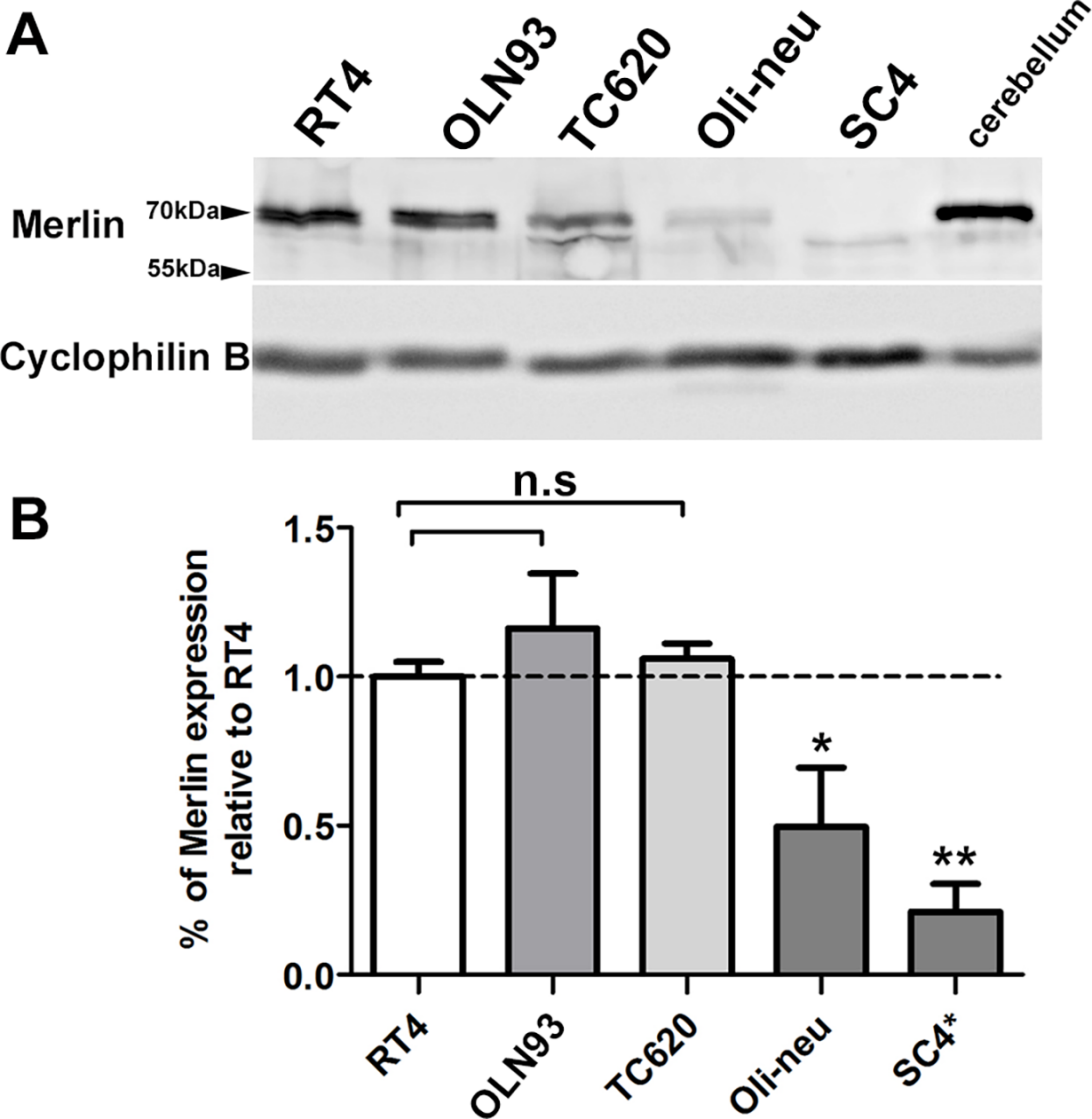
Quantification of Merlin expression in cerebellar tissue, cell lines and primary cell cultures. (A) Merlin expression is demonstrated by Western blotting loading 30 μg of total protein extract. Merlin was expressed as a 70kDa sized protein in RT4, OLN93, TC620 and Oli-neu cell lines; it was absent in SC4 cells. Western blots have been repeated three times with different samples and densitometrically evaluated (B). Expression levels have been related to the expression of Merlin in RT4 cells marked by the dashed line. Significant differences in expression of Merlin in oligodendrocyte cell lines vs. RT4 are marked by asterisks (mean ± SEM, n = 3; *** p < 0.001, ** p < 0.05 t-test).

To further support Merlin expression in oligodendroglial cell lines and in order to show full length protein expression, we used Western-blotting to compare amounts of Merlin expressed in oligodendrocyte cell lines to Merlin levels present in cerebellar extracts (Fig 3). Merlin appears as a 70kDa sized band in Western blots, and was detected in all cell lines and in mouse cerebellar extracts. However, the 70 kDa band of Merlin was absent in SC4 cells in which Merlin is more prominently degraded due to a deletion of exon 2 and 3 [51,52]. Instead of a 70 kDa band, a smaller and weaker band was apparent probably corresponding to an exon composition missing exons 2 and 3. Due to the deletion of exon 2 and 3, part of the FERM domain is missing in SC4-Merlin which is why SC4 cells are considered to be true Merlin-deficient cell lines [35,19]. In all cell lines the amount of Merlin expressed is lower than in the cerebellum, suggesting a rather low expression in oligodendrocytes. In addition to the 70kDa Merlin band, additional bands were detected which might represent different phosphorylation states or splice variants. These alternating bands were especially prominent in TC620 cells, the only human oligodendroglial cell line investigated here.

### Expression of Merlin isoforms in oligodendrocytes

**Fig 4:**
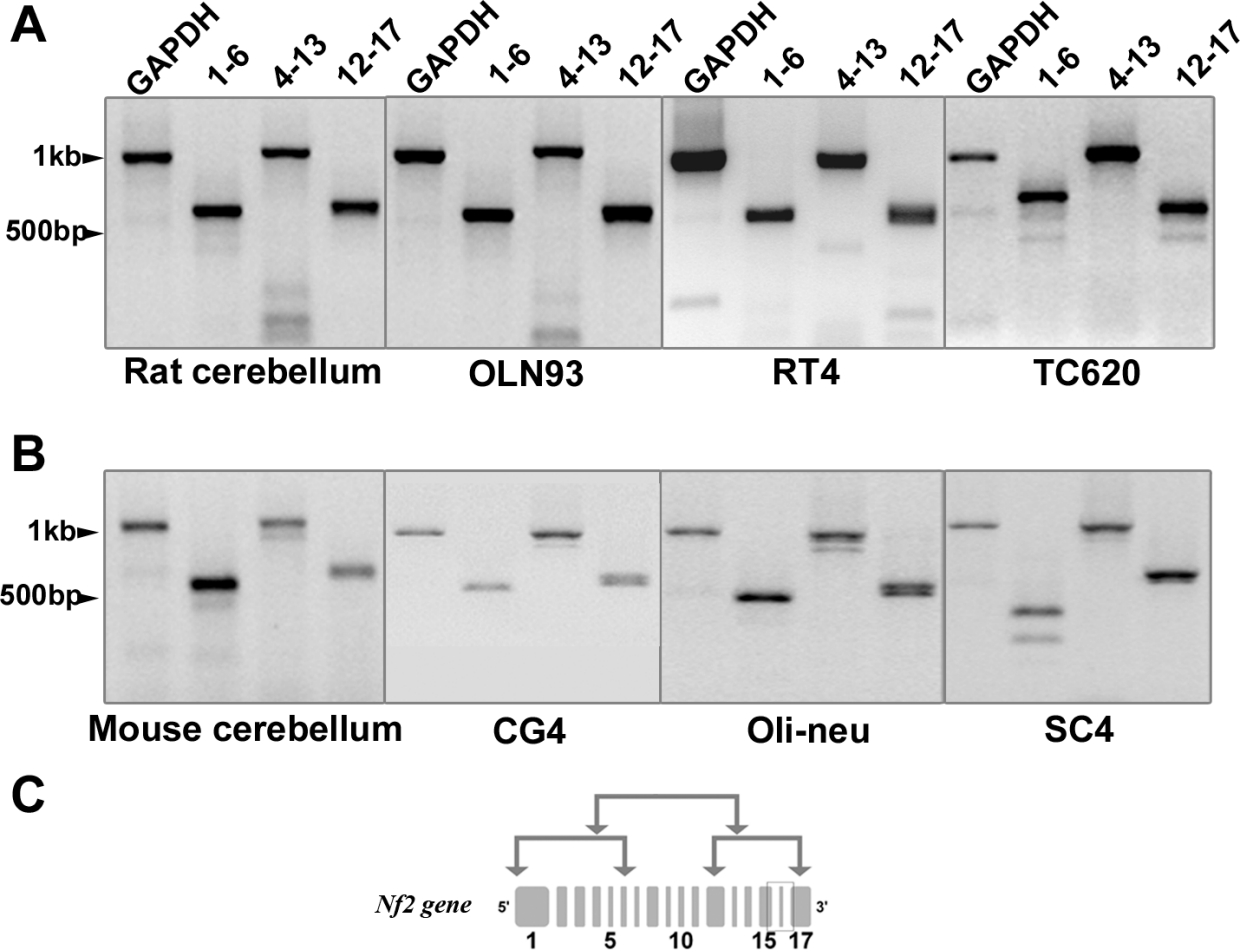
PCR analysis of Merlin isoform expression in cerebellar tissue and in cultured glial cells. Merlin isoform expression was analyzed by RT-PCR using three sets of primers spanning exons 1–6, 4–13 and 12–17 (see scheme in C). Primer sequences for rat (OLN93, RT4), human (TC620) and mouse (CG4, Oli-neu, SC4) Nf2 vary a little and result in slightly different fragment lengths (rat tissue and cells and human cell line in A, rat tissue and cells in B). Glyceraldehyde 3-phosphate dehydrogenase (GAPDH) expression was used as a control.

Considering the low amounts of Merlin protein found in oligodendrocytes, we decided to further prove Merlin expression by RT-PCR. In addition, PCR analysis allows us to identify splice variants of Merlin present in oligodendrocyte cell lines. It has been shown that two major and predominant isoforms of Merlin are generated by alternative splicing. This splicing event eliminates exon 16, which contains a stop codon; thus, a longer protein is translated (Isoform 1) [9]. Isoform 2 does contain exon 16; therefore, a shorter protein is translated from this mRNA. Other minor splicing variants have also been reported [3,21,53]. The scheme in Fig 4C shows the set of primers used.

In rat cerebellum, three fragments of 587, 1030 and 628 bps were amplified using primers for exons 1–6, 4–13 and 12–17, respectively (Fig 4A). The same fragment sizes were isolated from rat oligodendrocyte cell line OLN93 and rat schwannoma cell line RT4. In human oligodendroglioma cells TC620, three bands of 649, 1016 and 557 bp were amplified using the three sets of primers (Fig 4A). Accordingly, the expected fragments of 530 bp (exon 1–6), 1030 bp (exon 4–13) and 566 bp (exon 12–17) were amplified from mouse cerebellum, mouse oligodendrocyte primary cell cultures and from the mouse oligodendrocyte cell line (Oli-neu) (Fig 4B). These banding patterns suggest that Merlin isoform 1 mRNA is present in all cells analyzed and supports our protein data. However, besides isoform 1, multiple additional bands are apparent which differ from the expected band size by more than 100 bp suggesting the presence of splice variants which have not yet been characterized in these cell lines. E.g. in TC620 cells (Fig 4A), a band of around 500bp was observed when amplifying exons 1–6 and 12–17. These fragment sizes could result from elimination of exon 2 and exon 16. In the SC4 cell line, two fragments of 400 and 250 bp were amplified instead of a 530 bp fragment, resembling the elimination of exons 2 and/or 3. In addition, two separate bands could be visualized when amplifying exons 12–17 in oligodendrocytes primary cell cultures or Oli-neu cells. This could represent isoforms 1 and 2 which only differ by a 45 bp fragment. This isoform 2 might be present in other cell lines as well, given the rather thick band for exon 12–17 and the low resolution of the agarose gel (e.g. OLN93, RT4, and TC620). These results support the existence of *Nf2* mRNA in oligodendrocytes and the occurrence of different splice variants yet to be characterized.

### Effects of NF2 overexpression on oligodendrocyte cell lines

Merlin plays a crucial role in cell proliferation and in process outgrowth by changing the actin cytoskeleton dynamics [3,54]. As such, we analyzed whether manipulating Merlin expression in oligodendrocytes also affects cellular functions.

**Fig 5:**
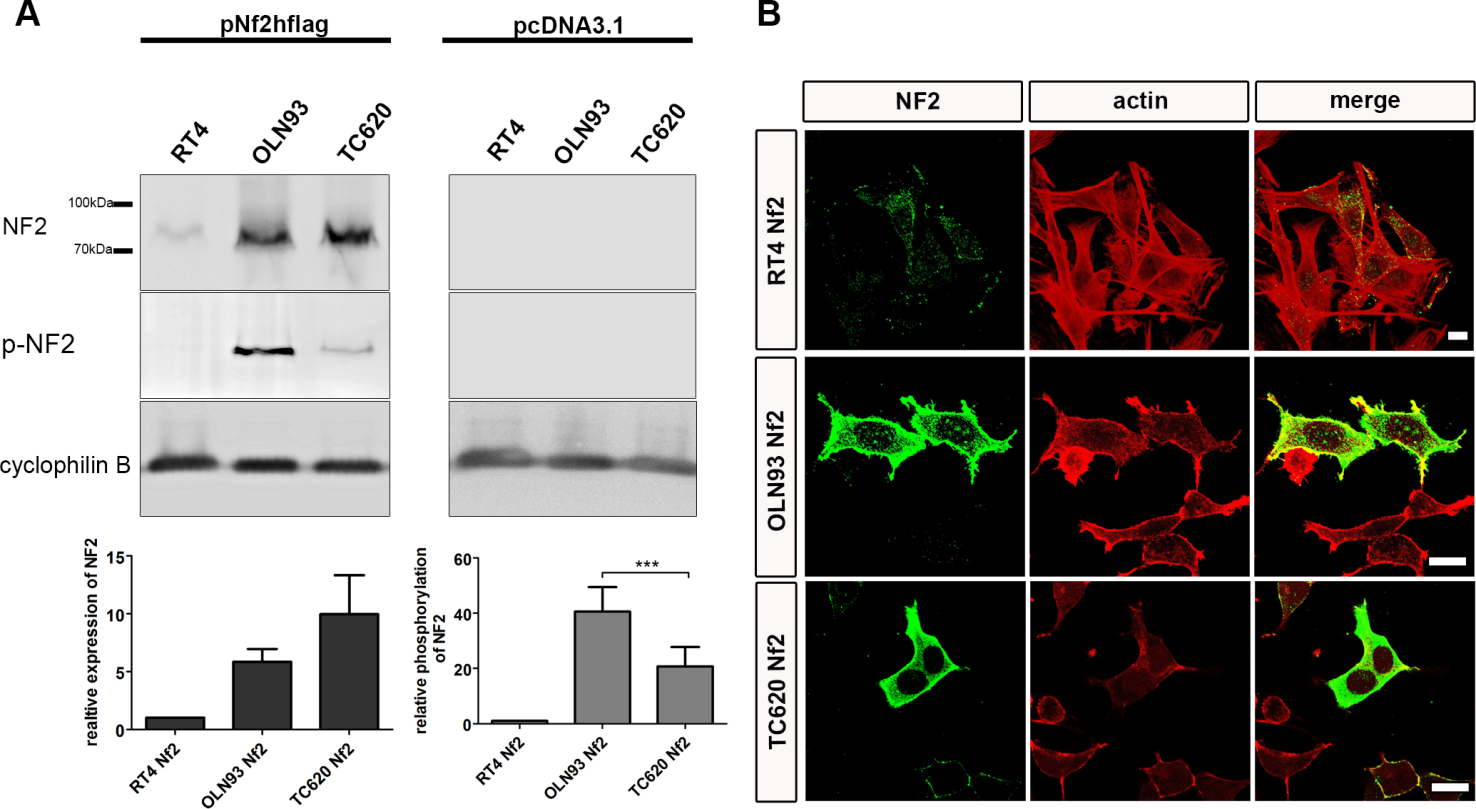
Expression of Merlin and phospho-Merlin in oligodendrocyte cell lines. RT4, OLN93 and TC620 cell lines were stably transfected with pcNF2hflag (Merlin) and pcDNA3.1 (control). Representative Western blots of gels loaded with 15 μg of total protein extracts are shown in A. The densitometric determination of Merlin and phospho-Merlin in the cell lines was performed by normalizing with cyclophilin B (mean ± SEM, n = 3, ***p < 0.001, Wilcoxon rank-sum test). Immunofluorescent staining of Merlin and phalloidin staining of actin in *Nf2* stably transfected cell lines demonstrates the preferred localization of Merlin in the cytoplasm (B). Single confocal planes of transfected cells are shown using the same microscope parameters. Scale bars: 10μm.

We stably transfected oligodendrocyte cell lines with a plasmid containing a neomycin resistance cassette and the human *Nf2* gene linked to a flag tag. After selecting the stably transfected cells with G418, we analyzed Merlin expression by Western-blot. *Nf2-flag* transfected oligodendroglial cells showed a prominent increase in Merlin expression, compared to cells transfected with the vector plasmid DNA pcDNA3.1 (Fig 5A). Please note that, in order to provide evaluable amounts of Merlin in *Nf2-*transfected cell lines, only 15 μg of total protein extracts were loaded; therefore, no Merlin expression could be observed in *control-*transfected cells. Base levels of Merlin expression have been shown in Western blots of Fig 3A for which 30 μg protein was loaded per lane. Since phosphorylation of Merlin at position S518 is considered to be a key step in the regulation of Merlin activity, we quantified the amount of phosphorylated *Nf2* in transfected cells. OLN93 transfected cells exhibited the highest level of phosphorylation; although they did not exhibit the highest amount of the Merlin protein when compared to the other cell lines (Fig 5A). In *Nf2-*transfected cell lines, Merlin was mainly concentrated in the cytoplasm, with only some signal detected in the nuclei (Fig 5B). When comparing the cellular distribution of Merlin in untransfected (Fig 2) and transfected cells, it is interesting to note, that Merlin localized to the nucleus was unchanged after transfection while the cytoplasmic content clearly increased. This might have consequences for the interpretation of the results which will be discussed. While all *Nf2*-transfected cells expressed Merlin, the level was highly divergent among cells. Some cells show high levels and some low levels of expression. However, there are also cells which appear unstained in the photomicrographs which are due to the fact that images were taken at an intensity which allowed us to see the cellular localization of Merlin.

### NF2 overexpression in oligodendrocytes affects cell proliferation and morphology

**Fig 6:**
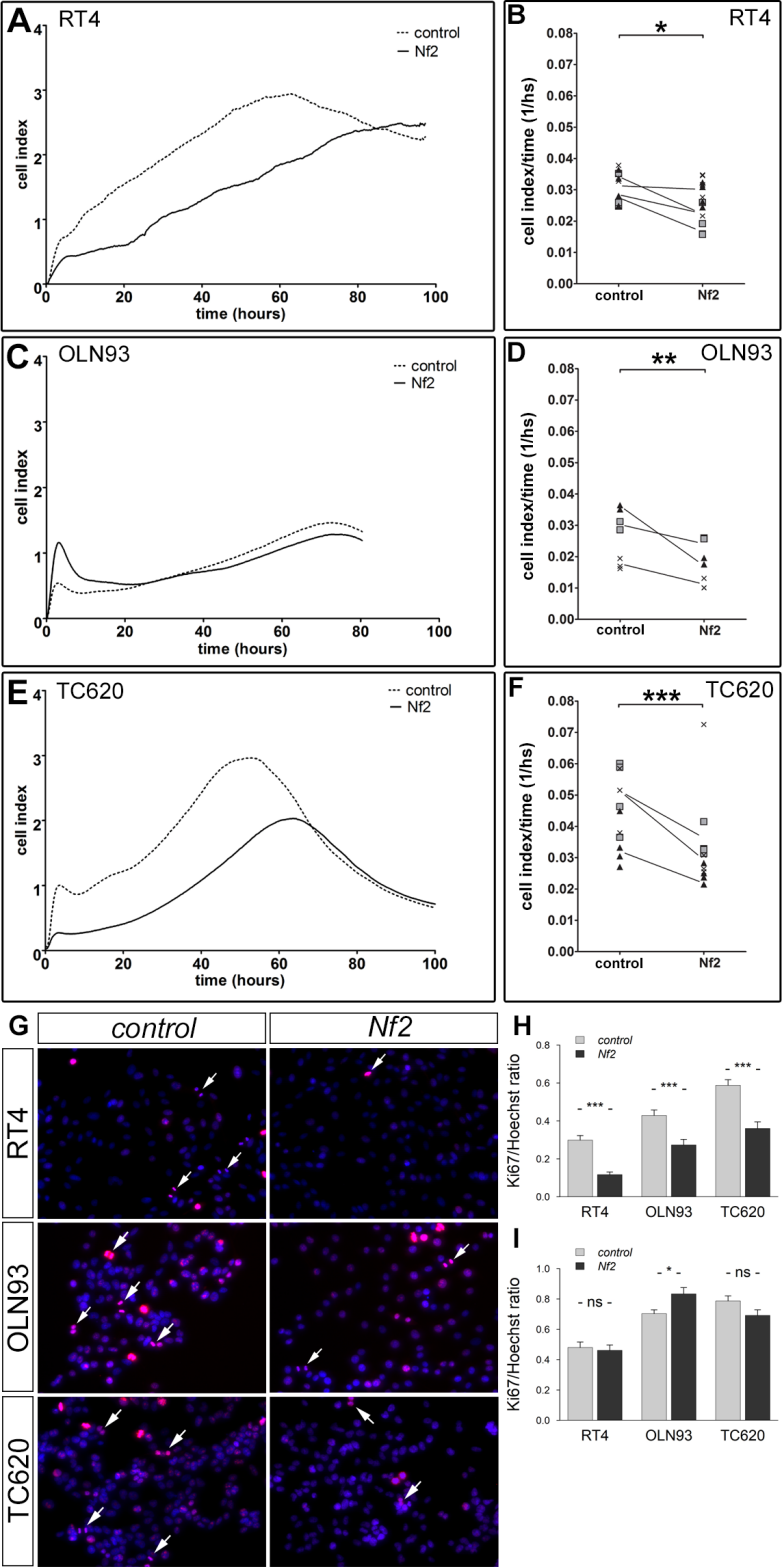
Merlin expression affects cell impedance in XCELLigence measurements. Cellular impedance was measured in RT4 (A, B), OLN93 (C, D) and TC620 cells (E, F), over-expressing *Nf2* and in control cells by XCELLigence. The cell index given as a measure of impedance of adhered cells, demonstrated a similar curve in all cell lines (A, C, E). Comparing cell indices over time by evaluating slopes of curves revealed a significant drop in *Nf2*-transfected (solid line) as compared to the *control-*transfected cells (dashed line) (B, D, F). Same symbols stand for replicates of one experiment. Lines connect corresponding average values of replicates within a single experiment. Significance values were calculated using the Wilcoxon signed rank test for independent samples (*p < 0.05; **p < 0.01; ***p < 0.001). Pictures in G represent typical images of Ki67 stainings (red). Mitotic figures are depicted by arrows. Total numbers of cells were visualized by Hoechst staining (blue, bar 10 μm). The relative Ki67/Hoechst staining was significantly decreased in *Nf2-*transfected versus *control*-transfected cells at 48 and more hours (H), but it was unchanged at shorter time periods (I, ns non-significant).

Schwann cells and some epithelial derived cells have been shown to suppress cell proliferation upon Merlin overexpression [55,56]. In a first approach to investigate Merlin´s role in oligodendrocyte proliferation and morphogenesis over time, changes in impedance were followed in NF2 overexpressing oligodendrocyte cell lines over a period of 100 hours. This was done using the biosensor *XCELLigence* method. This method measures the area of a cell culture dish covered by cells and detects changes in proliferation but also changes in morphology such as cell spreading. We tracked changes in impedance over four days, hypothesizing that initial changes in impedance might be due to adhesive properties of cells while long term effects more likely reflect changes in proliferation. A typical curve of cultured oligodendrocytes in XCELLigence (as it is for other cell types) was composed of an initial sharp increase in impedance (cell index), probably representing sedimentation and adhesion of cells (within 4–6 hours, Fig 6A, 6C and 6E). After adhesion, the cell index dropped slightly which could be caused by rounding of cells (within 8–10 hours). Then, the cell index increased continuously over a period of 50 to 60 hours depending on the cell line, and decreased again afterwards. Changes in the cell index were considered to be the slope of the curve between the initial settling phase—having taken place after 10 hours—and the maximum of the curve (Fig 6B, 6D and 6F). Three runs were conducted for each cell line, each with three replicates. All three cell types showed a significant reduction in the slope of the cell indices after NF2 overexpression. Reduction in cell indices over time amounted to about 20% in rat oligodendrocytes (OLN93), to 30% in human oligodendroglioma cells (TC620) and to 15% in schwannoma cells (RT4). In order to analyze whether changes in cell indices were associated to cell proliferation, we cultured cells for 96 hours and stained a batch every 24 hours. Evaluating the number of Ki67 stained pixels vs. the number of Hoechst stained pixels revealed a highly significant decrease in the Ki67/Hoechst ratio for all cell lines between 48 and 96 hours (Fig 6H). These data support a growth suppressive role of Merlin in oligodendrocytes and confirms data obtained for RT4 cells by other methods [57]. While Merlin overexpression seemed to reduce cell proliferation at longer time periods in culture, it only resulted in a slight increase in proliferation for OLN93 cells within 1 day after plating, but no change in the other cell lines (Fig 6I). The initial differences in Merlin overexpressing oligodendroglial cells as revealed by *XCELLigence* is therefore likely not due to proliferation, but rather to morphological changes.

### NF2 overexpression in oligodendrocytes affects process outgrowth

**Fig 7:**
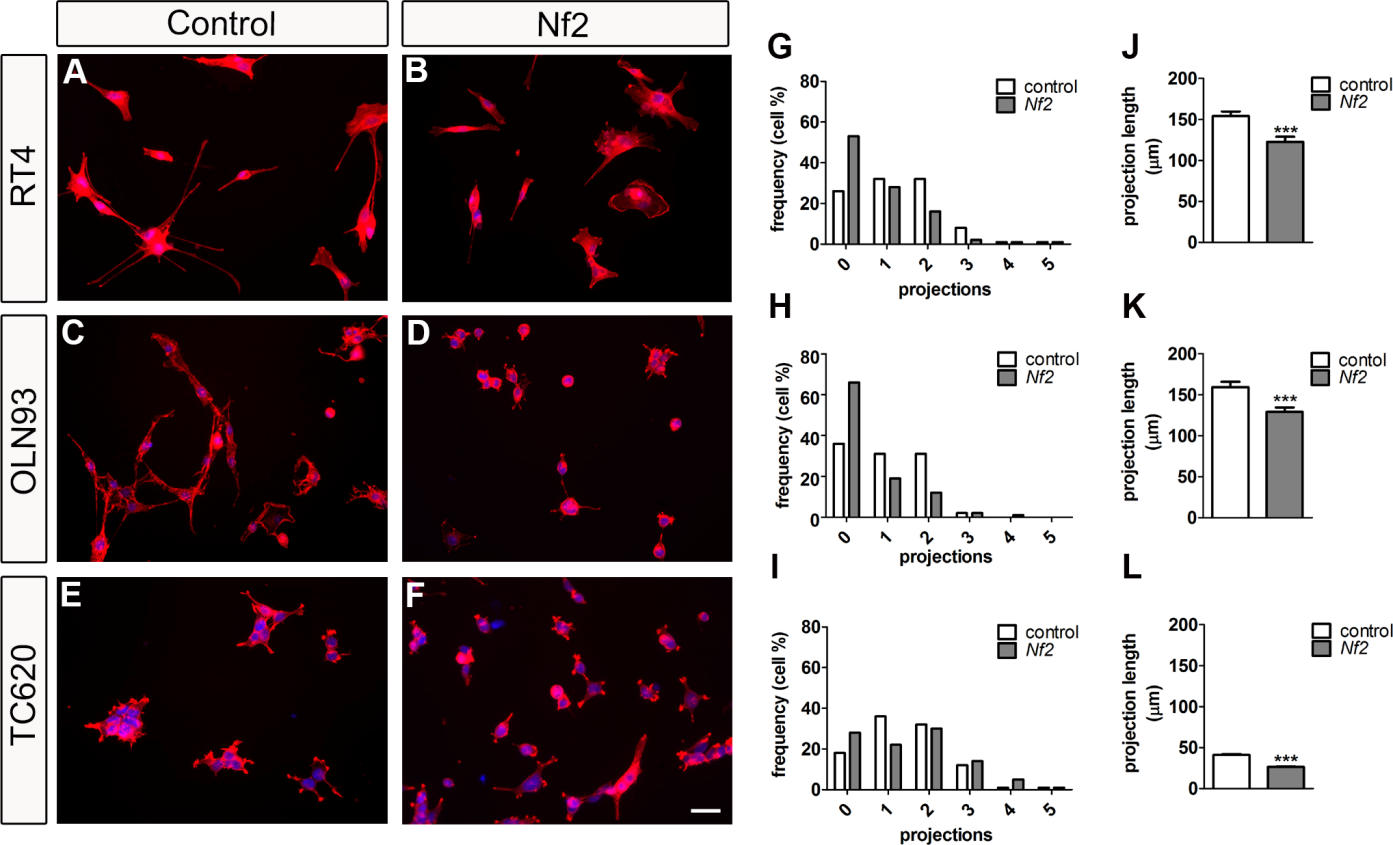
Merlin expression affects cell morphology. *Nf2*-transfected and *control vector-*transfected cell lines—RT4, OLN93 and TC620—were stained with phalloidin (actin cytoskeleton, red) and Hoechst 33342 (nuclei, blue) (A-F). Scale bar: 50 μm. Numbers of projections per cell are shown in G, H, and I as frequency distribution plots. Lengths of projections are shown in J, K, and L (mean ± SEM). *Nf2*-transfected RT4 and OLN93 cells contain significantly less processes as *control-*transfected cells (p < 0.01, Kolmogorov-Smirnov). Lengths of processes are significantly reduced in all cell lines (***p < 0.001, t-test; equal variances were tested by Levene’s test).

This morphological effect of NF2 expression was analyzed in more detail by measuring process outgrowth *in vitro*. Phalloidin staining of actin cytoskeleton showed dramatic changes in the cell shape and actin cytoskeleton organization in all transfected cell lines (Fig 7A–7F). Particularly, a significant decrease in the number of cellular projections in Merlin overexpressing cells was evident (Fig 7G–7I). While only 36% of the *control*-transfected OLN93 cells showed no process, 66% of Merlin overexpressing cells had no projections. Moreover, the percentage of OLN93 cells having one or two projections decreased significantly from 31% to 19% and from 31% to 12%, respectively (Fig 7H). Also, the length of the projections was significantly shorter in Merlin overexpressing OLN93 cells (129.13 ± 5.57 μm; n > 100) as compared to the *control-*transfected cells (159.53 ± 6.43 μm; n > 100, p < 0.001; Fig 7K). These results very much correspond to data which we obtained for rat schwannoma RT4 cells. Only 26% of control RT4 cells showed no projections, but 53% of Merlin overexpressing RT4 cells failed to build processes (Fig 7G). Again, the length of these projections was shorter in Merlin overexpressing RT4 cells (122.44 ± 6.32 μm; n > 100) as compared to *control*-transfected RT4 cells (154.12 ± 5.74 μm, n > 250; p < 0.001; Fig 7J). Although a tendency for fewer projections per cell in TC620 cells was visible, there was no significant difference. However, the length of these projections was significantly shorter in Merlin overexpressing (26.47 ± 0.81 μm; n > 400) as compared to the *control*-transfected TC620 cells (41.00 ± 1.18 μm; n > 1500; p < 0.001; Fig 7L).

These results clearly show that Merlin overexpression caused a reduction in process outgrowth, in number and in length. Furthermore, the inhibition of process elongation was directly related to the amount of Merlin expressed. Of the oligodendrocyte cell lines analyzed, TC620 cells expressed the highest amount of Merlin and exhibited the strongest inhibitory effect.

### NF2 overexpression reduces cell migration

A scratch assay was performed, in order to further support a function of Merlin in proliferation and migration. When a scratch is inflicted on a confluent monolayer, cells respond by closing the scratch through a combination of proliferation and migration.

**Fig 8:**
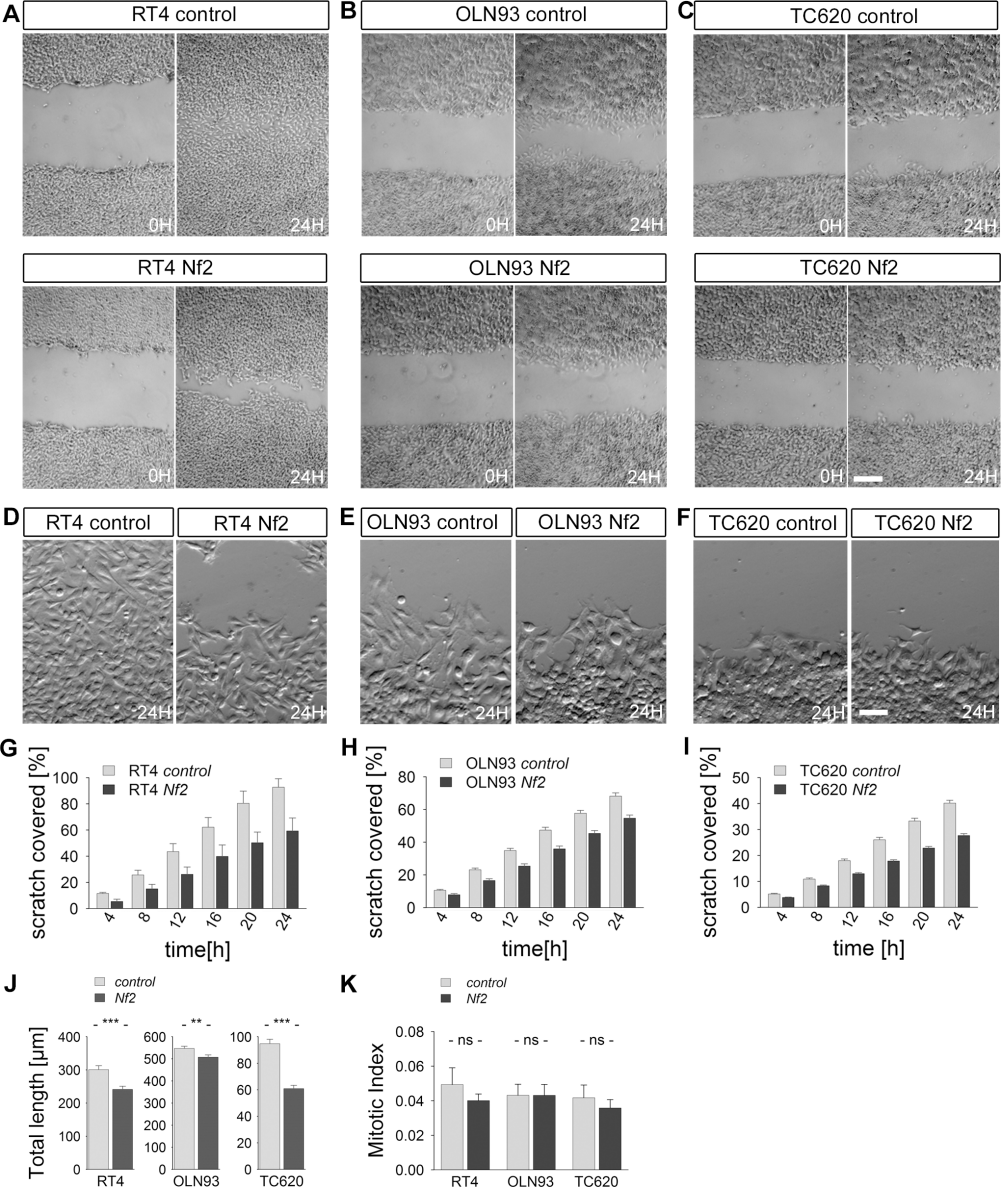
Merlin affects cell proliferation and migration in a scratch assay. Scratched areas are covered by new cells in cultures of RT4 (A, D, G), OLN93 (B, E, H) and TC620 (C, F, I) cell lines, stably transfected with pcNF2hflag (*Nf2*) and pcDNA3.1 (control). (A-C) Images show scratched areas in the culture at the time of scratching and 24 hrs. later. (D-F) To visualize cell morphology in scratched areas after 24 hrs. in culture, higher magnifications are shown. (G-I) Plots shown represent the percentage of the original cell-free area covered by cells after every 4 hours, during a 24 hour period (mean ± SEM, n = 4, *p < 0.05; **p < 0.01, Two-way ANOVA). (J) Cell tracking revealed a significant reduction in total path length between *Nf2*- and *control-* transfected cells (mean ± SEM, n_TC620_ > 150, n_OLN93_ > 200, n_RT4_ > 70, **p < 0.01, ***p < 0.001; Wilcoxon rank test). Please note that RT4 cells have run for only 10 hours due to their higher velocity, while OLN93 and TC620 have run for 30 hours. (K) The mitotic index is given as the number of phosphorylated histone H3 cells as compared to total number of cells. No significant difference could be observed between *control-* and *Nf2*-transfected cells. Scale bars: (A) 100 μm, (B) 25 μm.

*Control-*transfected RT4 cells in culture were able to successfully close the denuded area 24 hours after the scratch was inflicted (92.7 ± 6.5% of the cell-free region was closed). In contrast, *Nf2-*transfected RT4 cells were not able to completely close the cell-free region (only 59.3 ± 9.9% of the cell-free regions was covered with cells, Fig 8A and 8G). RT4 cells bordering the scratch displayed long processes and spindle like structures oriented towards the scar, demonstrating a typical feature of migrating cells (Fig 8D). In addition, we could show that the mitotic index in RT4 cells was not significantly changed within the scratched area after and during refilling the scratch (Fig 8K). A similar result could be obtained for OLN93 and TC620 cells. OLN93 cells closed the scratch by 37.8 ± 5.2% when transfected with a control vector and they filled up the scratch by 23.1 ± 2.9% when transfected with *Nf2* (Fig 8B and 8H). A comparable difference in migration could be observed between *control-*transfected and *Nf2-*transfected TC620 cells (25.2 ± 2.1% TC620_*control-*transfected_ vs. 17.8 ± 2.5% TC620_*Nf2-*transfected_; Fig 8C and 8F). Consistently, OLN93 and TC620 *Nf2-*transfected cells did not show differences in the mitotic index during the culture period (Fig 8K).

In order to dissect out the relevance of migration versus proliferation in scratch assays, we performed time lapse imaging and tracked single cells. Since the variability is rather high from cell to cell, we selected for those cells which were not encased by other cells during the first 10 hours, and which could be followed for at least 25 hours. If a cell divided during the migration path, only one of the daughter cells was followed. As shown in Fig 8J, all *Nf2*-transfected cell lines showed a significant reduction in path length within 30 hours as compared to control cells. This difference was most prominent in TC620 cells (40% reduction); a 25% reduction could be observed in RT4 cells and a 10% reduction in OLN93 cells. None of the cell lines showed a difference in the mitotic index between Merlin over-expressing and control cells during the 30 hour period investigated, although some tendency towards reduced mitosis might be adumbrated after 24 hours. Taken together, we conclude that NF2/Merlin, besides impacting on mitotic activity, affects movements of oligodendrocyte cell lines.

## Discussion

The neurofibromatosis type 2 gene is mainly investigated in the peripheral nervous system for its function as a tumor suppressor. In recent years, its role in the central nervous system gained increasing attention. While present in neurons and astrocytes [7,9,28,46,24], little is known about its expression and function in the myelinating cells of the central nervous system. Here, we demonstrate that NF2 is expressed in developing and mature oligodendrocytes at low, but considerable levels *in vivo* and *in vitro*. Merlin overexpression in oligodendroglial cell lines reduced proliferation and diminished morphological differentiation and migration.

Allocation of Merlin expression to distinct cell types of the central nervous system through immunohistochemistry requires a selective antibody with solid affinity to NF2, and a highly sensitive procedure to detect the target protein. In this and previous studies, we successfully used NF2 (A19) and NF2 (C18) antibodies from Santa Cruz with comparable results [3,4]. Our staining procedure did not stain forebrain or cerebellar tissue when omitting the first antibody. In addition, the antibody-associated staining could be blocked by peptides presenting the antibody epitope [3]. Unconditioned knockout mice which are unable to express NF2 in any tissue do not survive; this prevents their use as a control tissue. Isoform specific knockout mice still contain one isoform which is recognized by the antibodies used [4]. When overexpression NF2, we could clearly demonstrate an increase of a unique band running at the appropriate size in Western-blots supporting the specificity of the antibodies used here. Further support for the functionality of the antibodies comes from other laboratories that used these antibodies in immunofluorescence and Western blot studies (e.g. [58,59]).

In addition to our immunofluorescent analysis, we have also proven the existence of Merlin in oligodendrocytes *in vitro* by PCR and Western blotting. Interestingly, by using three different primer pairs spanning overlapping parts of *Nf2*, we could detect multiple amplicons, suggesting that *Nf2* exists as more than two splice variants in oligodendrocytes. Besides the full length *Nf2* transcript, a somewhat shorter amplicon could be detected using primers binding to exons 12 and 17. According to its size, this *Nf2* DNA fragment most probably represents isoform 2, which displays a functionally divergent version having a shortened 3’ terminus [60,61]. In both human TC620 oligodendroglioma cells and in mouse cerebellum, additional shorter DNA fragments were consistently amplified using primers binding to exon 1 and 6. These might represent deletions of exon 2 and/or 3 [53]. So far, additional amplicons derived from primer pair 4 and 13 are less well attributable to known splice variants, but may represent deletions of exon 6 to 9, previously described as isoform 10 [21]. The major point we want to make here is that PCR analyses support our observation of Merlin to be expressed in different oligodendrocyte cell lines. The rather low expression level in oligodendrocytes, when compared to neurons and astrocytes, sustains a previous observation [7].

Analysis of Merlin expression revealed its presence in oligodendrocytes throughout cellular differentiation. Merlin immunosignal co-localized with Olig2, which precedes platelet-derived growth factor receptor alpha expression in the oligodendrocyte lineage and is routinely used as a marker of young oligodendrocytes in the cerebellum [62–64]. In addition, Olig2 and Merlin expressing cells were found in the white matter of the cerebellar cortex at a time when no mature oligodendrocytes are present. In adult mice, Merlin co-localized with myelin basic protein, a classical marker of mature oligodendrocytes [65]. In addition, we could find Merlin expression in cultured oligodendrocytes which also express the proteoglycan NG2. NG2-positive cells are considered to be glia precursor cells being able to generate oligodendrocytes [66]. We thus conclude that Merlin is expressed from early precursor cells and continuing into later oligodendrocyte cell stages.

The spatiotemporally defined expression pattern of Merlin suggests multiple roles of Merlin in oligodendrocyte maturation. The *Nf2* gene was initially characterized as a tumor suppressor gene. Merlin’s link to the control of cell proliferation is evidenced by the fact that human schwannomas, meningiomas and mesotheliomas usually exhibit a loss of both *Nf2* alleles [54,67–70]. Consistently, a reduction of Merlin expression in schwannoma cell lines using antisense oligonucleotides led to a 1.3 fold increase in cell proliferation [71]. Besides, Merlin overexpression in NIH 3T3 cells leads to a decrease in the cell growth rate, reinforcing its role as a tumor suppressor [72]. Here we could show that *Nf2* overexpression in OLN93 and TC620 cells caused a slower increase in the cell index as obtained by XCELLigence measurements. This increase in cell index was observed over a time period of up to 100 hours and was previously suggested to be due to proliferation [73]. Although this is clearly not the case for the first 24 hours as shown by Ki67 stainings, NF2 overexpression significantly reduced oligodendrocyte proliferation at later time points. This decrease in proliferation by NF2 overexpression well corresponds to findings in Schwann cells [13,19]. Changes in responsiveness of cultured cell lines to Merlin overexpression might have several reasons. First, we could show that TC620 cells expressed higher amounts of Merlin as compared to OLN93 and especially RT4 cells. Gene dosage effects are well known for Nf2 since loss of heterozygosity in NF2 patients often causes tumor formation and/or polyneuropathies [74,75]. In addition, cell context dependent effects might occur. The cell lines used in the present study have been immortalized by different genetic manipulations [33,57] which might affect cell environment. These differences in cell environment might, for example, cause variations in Merlin phosphorylation status over time, which affects its function [76]. Indeed, we could show that the level of Merlin phosphorylation on S518, commonly related to Merlin inactivation, was variable in between cell lines. The phosphorylation status of a cell is dependent on various signal transduction pathways and it is known that Merlin participates in many signaling pathways such as Wnt and RhoA and the stimulation of the Lats2 pro-apoptotic pathway [31]. An additional reason for cell-type specific and time dependent variations in Merlin activity might be its localization and isoform specific cellular distribution. The nuclear to cytoplasmic distribution was higher in untransfected TC620 cells as compared to OLN93 and RT4 cells. After overexpressing isoform 1 the cytoplasmic fraction of Merlin increased dramatically while the nuclear fraction remained unchanged. The relative amounts of isoforms present and their distribution to distinct cellular compartments might influence Merlin’s function. Without being able to dissect apart the different possible mechanisms at this time, we can conclude that cytoplasmic Merlin can inhibit proliferation of oligodendrocyte cell lines as was shown for other glial cell lines. Hence, Merlin integrates different signaling pathways for cell proliferation control. It is therefore plausible that Merlin affects proliferation in a cell type specific and time dependent manner.

While Merlin’s role in tumor progression and cell proliferation has been investigated more rigorously and could be supported here, its effect on process outgrowth is less well known. In all oligodendroglial cells analyzed, Merlin overexpression decreased the number and the length of cellular processes in a cell context dependent manner. In addition, Merlin overexpression caused a slower rate of migration in a culture based scratch assay. Re-filling of scratched areas might occur by proliferation; however, no difference in mitotically active cells could be observed between *control-* and *Nf2-*transfected cells. Instead, NF2 overexpressing cells moved shorter distances. This suggests a major contribution of cell movements to covering cell-free areas during the time investigated. These results are consistent with previous findings showing that reduced Merlin expression levels lead to an increase in tumor cell migration [77–79]. Both, proliferation and process growth regulation by Merlin can be mediated by a variety of signaling pathways which includes Rac–PAK signaling, the PI3K/Akt/mTOR pathway, FAK–Src activation [18], Hippo-YAP signal transduction [80,81] or inhibition of the nuclear E3 ubiquitin ligase CRL4^DCAF1^ [82,83]. Several upstream regulators have been identified which stimulate these signaling pathways in a Merlin dependent way. E.g. Merlin was shown to physically interact with syntenin, which binds the oligodendroglial precursor protein NG2 [84,85]. Epidermal growth is suppressed by Merlin mediated regulation of regulated tyrosine kinase and epidermal growth factor receptor activity [56,86]. Human mesotheliomas overexpressing Merlin reduce its proliferation rate by down-regulating cyclin D1 expression through PAK signaling pathway [87]. Many of these pathways are active and essential for oligodendrocyte differentiation [88–94]. It is thus also mechanistically plausible that Merlin does indeed affect process formation in oligodendrocytes. The neurofibromatosis type 2 product might therefore impact on neuron-glia interactions in the central nervous system as it does in the peripheral nervous system [95].
